# Prevalence, type and concentration of human enterovirus and parechovirus in cerebrospinal fluid samples of pediatric patients over a 10-year period: a retrospective study

**DOI:** 10.1186/s12985-015-0427-9

**Published:** 2015-11-25

**Authors:** Silke Vollbach, Andreas Müller, Jan Felix Drexler, Arne Simon, Christian Drosten, Anna Maria Eis-Hübinger, Marcus Panning

**Affiliations:** Institute of Virology, University of Bonn Medical Centre, Sigmund-Freud Str. 25, 53105 Bonn, Germany; Department of Paediatrics, University of Bonn Medical Centre, Sigmund-Freud Str. 25, 53105 Bonn, Germany; Institute of Virology, Medical Center - University of Freiburg, Hermann-Herder Str. 11, 79104 Freiburg, Germany; Present address: Paediatric Oncology and Haematology, University of the Saarland, Homburg, Germany

**Keywords:** Human parechovirus, Enterovirus, Cerebrospinal fluid, Prevalence, Viral RNA concentration

## Abstract

**Background:**

Human enterovirus (EV) and parechovirus (HPeV) are significant causes of encephalitis and meningitis in children. The aim of this study was to determine the prevalence, type and viral RNA concentration of EV and HPeV in cerebrospinal fluid (CSF) samples in an unselected cohort of patients <18 years admitted to Bonn university hospital from 1998 to 2008.

**Methods:**

A total of 327 CSF samples from 327 patients were retrospectively tested by real-time reverse-transcriptase PCR (RT-PCR) for EV and HPeV, and by real-time PCR for cytomegalovirus (CMV), herpes simplex virus 1/2 (HSV), and varizella zoster-virus (VZV). Samples had been submitted for routine virological work-up due to suspected meningitis or encephalitis and had been stored at −20 °C hereafter. Positive samples for EV and HPeV were sequenced within the gene encoding the VP1 region (EV), the VP2 and the VP3/VP1 junction region (HPeV).

**Results:**

The overall prevalence was 4.3 % (14/327) for EV, 0.6 % (2/327) for HPeV, and 0.3 % (1/327) for HSV and VZV, respectively. CMV was not detected in this cohort. In children less than 3 months of age the prevalence was 7.7 % (2/26) for EV and 7.7 % (2/26) for HPeV, respectively. Frequency of EV detection ranged from 0 to 12 % per year and highest rates were observed from June to September. All typed EV belonged to species B. Both HPeV infections were detected in the fall of 2008 and were typed as HPeV genotype 3. Viral RNA concentrations were highest in patients with HPeV infection, followed by echovirus 30 and other EV. In total, 86 % (12/14) of EV infections presented as aseptic meningitis, whereas both HPeV infections presented as severe sepsis-like illness.

**Conclusions:**

EV and HPeV were equally prevalent in children <3 months of age. Beyond the detection of EV and HPeV, the determination of viral RNA concentration and typing of EV and HPeV might prove beneficial for patient management and public health.

## Background

Human enteroviruses (EV) and parechovirus (HPeV) belong to the highly diversified family *Picornaviridae* and constitute an important cause of central nervous system (CNS) infections, meningitis and encephalitis, and sepsis-like illness in children worldwide [1–3]. EV can be grouped into 4 genetically distinct species, EV-A to D. The most commonly detected EV associated with meningitis belong to EV species B (including echoviruses, coxsackievirus (CV)-A9, and CV-B). Continued surveillance and monitoring of EV evolution is important not only for public health (EV disease association, identification of novel variants), but also for patient management (isolation measures, treatment with IVIG). In 1997 an EV-A71 sparked an ongoing epidemic across the Asia-Pacific region and is associated with hand, foot, and mouth disease accompanied by severe neurological complications [4]. Recently, an EV-D68 emerged and caused considerable morbidity and mortality among children worldwide [5, 6]. For HPeV at least 16 genotypes have been described to date, most of them only recently (www.picornastudygroup.com). In particular, HPeV genotype 3 (HPeV3) infections tend to be more severe (including sepsis-like illness) compared to infections with other HPeV genotypes [7].

The mainstay of laboratory diagnostics for EV and HPeV infection depends on real-time reverse-transcription-PCR (RT-PCR), which has considerably improved the detection of EV as well as HPeV [8]. However, HPeV infection is still reported infrequently and is most likely under-diagnosed in Germany and elsewhere. Information on the epidemiology over longer time periods and virus concentrations for EV and especially HPeV in cerebrospinal fluid (CSF) samples is lacking at large.

The aim of this study was to determine the prevalence, types and RNA concentrations of EV and HPeV in an unselected panel of CSF samples collected from pediatric patients with a clinical suspicion of meningitis/encephalitis or late-onset sepsis with neurological symptoms admitted at a large university hospital over a 10-year period to assess the role of these pathogens in pediatric disease.

## Results

Median patient age was 8.0 years (range, 8 days to 17 years). Eight percent of patients (26/327) were under the age of 3 months. An overall prevalence of 5.5 % (18/327) for any viral pathogen was determined by real-time reverse-transcriptase PCR (EV, HPeV) and real-time PCR (CMV, HSV, VZV), respectively. EV was the most prevalent pathogen with 3.4 % (11/327) using the assay described by Verstrepen et al. [9]. Upon use of the assay by Dierssen et al. [10] prevalence increased by a quarter to 4.3 % (14/327) (9 male, 5 female). In children less than 3 months of age 7.7 % (2/26) tested positive for EV with both EV assays (Verstrepen et al. and Diessen et al.) yielding congruent positive results. For HPeV an overall prevalence of 0.6 % (2/327) was determined (one female, one male). Both HPeV RT-PCR assays applied yielded congruent results. If children older than 3 months of age were excluded from the analysis, the prevalence of HPeV raised to 7.7 % (2/26) similar to that of EV. One individual patient was positive for HSV-1 (6 year-old male) and another for VZV (4 year-old male) as determined by real-time PCR yielding an overall prevalence of 0.3 % for each virus. Clinical information was lacking for these two patients. Cytomegalovirus was not detected (0/327).

**Fig. 1:**
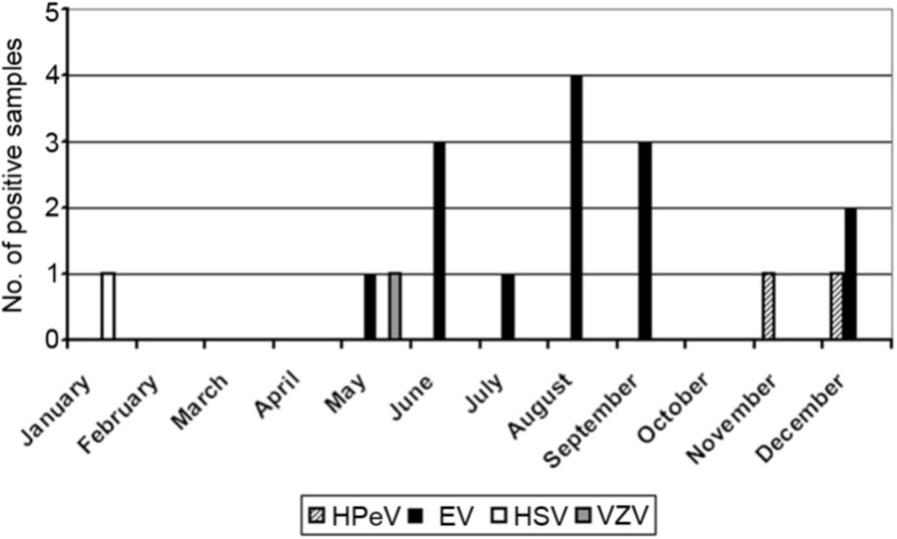
Monthly distribution of positive samples. Shown is the cumulative number of positives samples in each month from January to December. Each viral pathogen detected (HPeV, EV, HSV, VZV) is indicated by a different bar

Since a seasonal pattern of occurrence for EV and HPeV has been described cumulative detection rates were plotted from January through December (Fig. 1). For EV highest rates were observed in the summer months from June to September. No EV detections were seen in the years 1998, 1999, 2003 and 2006 (Table 1). The two HPeV cases were detected in October and November 2008, respectively. The HSV positive sample was detected in January 2008 and the VZV positive one in May 2007.

**Table 1:** Proportion of samples positive for EV, HPeV, HSV, and VZV in CSF samples collected from 1998 to 2008

		Proportion (%) of virus-positive samples			
Year	Number of patients	EV	HPeV	HSV	VZV
1998	11	0	0	0	0
1999	12	0	0	0	0
2000	27	3 (11.1)	0	0	0
2001	27	1 (3.7)	0	0	0
2002	31	1 (3.2)	0	0	0
2003	23	0	0	0	0
2004	24	3 (12.5)	0	0	0
2005	34	1 (2.9)	0	0	0
2006	42	0	0	0	0
2007	47	2 (4.2)	0	0	1 (2)
2008	49	3 (6.1)	2 (4.1)	1 (2)	0
Total	327	14 (4.3)	2 (0.6)	1 (0.3)	1 (0.3)

To further characterize the EV positive CSF samples the VP2 region was partially sequenced. All EV for which sequencing was successful (*n* = 10) belonged to EV species B. In detail, six samples were echovirus 30, one was echovirus six, one echovirus 13, one CV-B3, and one was CV-B6. Of note, in one patient in whom typing of the CSF sample was unsuccessful, EV-30 RNA could be identified in a corresponding stool sample. This sample was rated as EV-30 in all subsequent analyses. All EV-30 positive samples were randomly scattered throughout the entire study period. A total of three samples with EV concentrations of 3.3, 3.8, and 5.9 log10 RNA copies/ml, respectively, remained refractory to typing. Sequencing failed in CSF samples from two patients due to rather low RNA concentrations. In the sample with the highest viral RNA concentration no leftover impeded typing. Sequencing of the VP1/VP3 region of the HPeV positive samples yielded HPeV3 in both cases. Both HPeV3 samples lacked a RGD motif in the VP1/2A junction region.

In a next step, virus RNA concentrations of EV and HPeV in CSF samples were analyzed. The mean virus concentration of the EV positive samples was 4.3 log10 RNA copies/ml [95 % confidence interval (CI) 3.9–4.7 log10 RNA copies/ml]. For further analysis two groups of EV-positive samples were compared. One group comprised samples with EV-30 (*n* = 7) and the second group all other non EV-30 EV samples (*n* = 4) in which typing was successful. Mean virus concentration in the first group was 4.4 log10 RNA copies/ml, in the latter group 4.2 log10 RNA copies/ml (Mann-Whitney U-test, *p* >0.05).

**Fig. 2:**
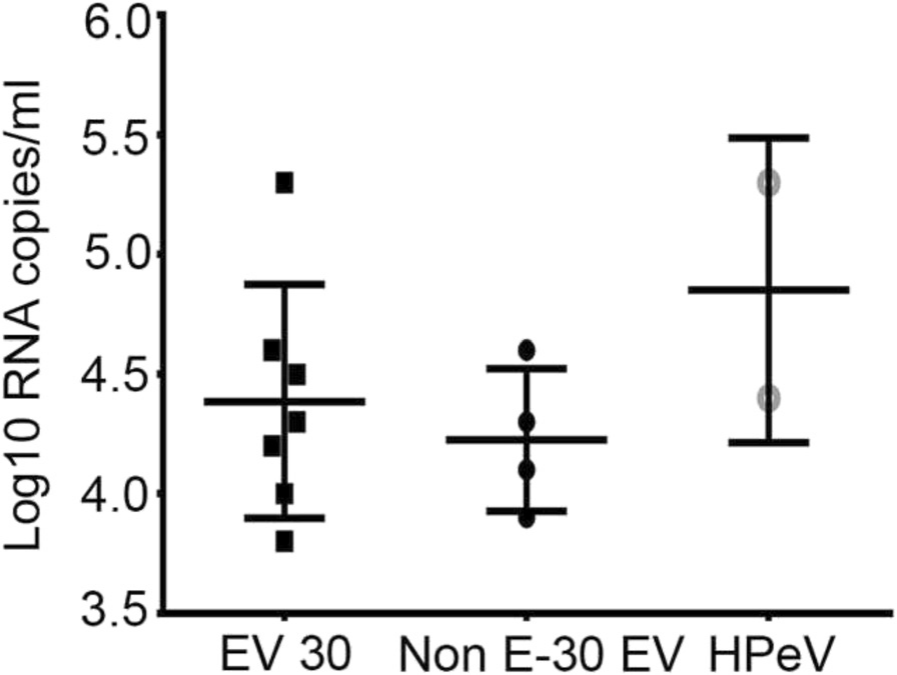
Viral RNA concentrations. Log10 RNA concentration per ml CSF in samples positive for echovirus 30, non E-30 EV, and HPeV, respectively. Virus concentrations are given on a log scale on the y-axis. Each dot represents 1 specimen. Horizontal lines represent median values for each sample group. Outer horizontal lines represent the 95 % CI

An HPeV RNA concentration of 5.3 log10 RNA copies/ml was measured in a 5-week-old child and a concentration of 4.4 log10 RNA copies/ml in a 1-week-old neonate, respectively. Next, viral loads between echovirus 30 (*n* = 7), all other typed non EV-30 EV positive samples (*n* = 4) and both HPeV positive samples were compared. Mean log10 viral RNA concentrations of EV-30, other typed non EV-30 EV, and HPeV (4.9 log10 RNA copies/ml) samples were not significantly different as assessed by one-way ANOVA (Fig. 2). Of note, mean interval between symptom onset and drawing of CSF was 0.4 days for EV positive samples and 4.5 days for HPeV positive samples (*p* = 0.002, two-tailed t-test). Of note, in the 5-week-old child with HPeV infection a second lumbar puncture was performed on day 7 after symptom onset upon clinical deterioration (periodic breathing, lack of spontaneous movement). The first lumbar puncture was performed on day 1 of symptoms (hypothermia, sucking weakness, and reduced general condition) but CSF was used up for other testing. Mean virus concentrations of typed EV (*n* = 2) and HPeV (*n* = 2) in children <3 months of age were not significantly different (4.5 log10 RNA copies/ml versus 4.9 log10 RNA copies/ml, *p* <0.05, two-tailed t-test).

Mean age of children with EV-30 infection was 5.1 years (range, 1–9), 1.6 years (range, 3 months-6 years) for those with non EV-30 EV infection, and 3 weeks for those with HPeV3 infection. Aseptic meningitis was diagnosed in 12/14 (86 %) patients with EV infection, but not in patients with HPeV infection (Table 2). The indication for lumbar puncture (i.e. aseptic meningitis or encephalitis or other neurological symptoms) could not unambiguously be retrieved for one child with EV infection, for another child with EV infection fever, headache, meningism without pleocytosis was recorded. No clinical signs of encephalitis were reported in those with EV infection, but in one child with HPeV infection. Clinically, both infants positive for HPeV displayed sepsis-like illness. In the 1-week-old neonate this included reduced general condition, sucking weakness, fever, and on day 2 tonic-clonic seizures. Cranial ultrasound showed hyperechogenicity, slurred gyrification, and hyperemia. The 5-week-old infant first presented with hypothermia, sucking weakness, reduced general condition, rash, and later developed respiratory distress. No fatal outcome was observed in the entire study cohort.

**Table 2:** Proportion of clinical characteristics between patients with EV, echovirus 30, non EV-30 EV, and HPeV in CSF samples

Characteristic	All EV (*n* = 14)	Echovirus 30 (*n* = 7)	Non EV-30 EV (*n* = 4)	HPeV (*n* = 2)
Fever/Hypothermia	93 % (13/14)	86 % (6/7)	100 % (4/4)	100 % (2/2)
Meningism	57 % (8/14)	71 % (5/7)	25 % (1/4)	0 % (0/2)
Headache	57 % (8/14)	86 % (6/7)	25 % (1/4)	na
Photophobia	21 % (3/14)	29 % (2/7)	0 % (0/4)	na
Sepsis-like illness	0 % (0/14)	0 % (0/7)	0 % (0/4)	100 % (2/2)
Aseptic meningitis	86 % (12/14)	86 % (6/7)	100 % (4/4)	0 % (0/2)
Encephalitis	0 % (0/14)	0 % (0/7)	0 % (0/4)	50 % (1/2)
Respiratory tract symptoms	50 % (7/14)	57 % (4/7)	25 % (1/4)	50 % (1/2)
Gastrointestinal symptoms	64 % (9/14)	86 % (6/7)	50 % (2/4)	0 % (0/2)
Rash	0 % (0/14)	0 % (0/7)	0 % (0/4)	50 % (1/2)

*na* not applicable

## Discussion

In our cohort, we could show that HPeV3 is equally prevalent to EV in infants less than 3 months of age, which has not been observed in other studies so far. Previous studies showed a prevalence of HPeV in CSF samples ranging from 0 to 17 % [11, 12]. However, studies differed by inclusion criteria, study design, and demographic characteristics. In our study we used an unbiased approach and included all CSF samples which were sent from the pediatric department for routine diagnostics. Walters et al. used a similar approach and reported a prevalence of 2.3 % for HPeV among children <3 months of age, which is in close agreement to our results [2]. In contrast to other studies, we did not observe a 2-year seasonality for HPeV [3]. A different epidemiology in Germany cannot be ruled out but the samples size per year was rather small and prolonged storage may have influenced our results. This is supported by another study from our laboratory where other HPeV types were more frequently detected in respiratory tract and stool samples compared to HPeV3 [13].

Of note, both HPeV cases occurred in the fall of 2008, which contrasts with a report from another European country, where most infections were seen in the spring [11]. Recent data from the USA indicate that the peak season for HPeV detection was in the summer months [12]. The highly variable epidemiology of HPeV is supported by recent findings from South Korea, where the majority of cases were detected in spring in one study and in summer in another study [14, 15]. However, both studies covered only a short period of time. Beyond the detection of HPeV in clinical samples using PCR seroepidemiological data is urgently needed to better appreciate the epidemiology and impact of HPeV in general and of HPeV3 in particular. As expected for EV we observed a peak of cases in the summer months.

Typing of HPeV samples showed exclusively HPeV3, which is consistent with other studies. In contrast, typing of EV samples showed a diversity of EV species B subtypes. A seroepidemiogical study from France demonstrated the predominance of EV species B from 2000 to 2004 and echovirus 30 was the most frequently detected EV, which is supported by our data [16]. Noteworthy, similar findings were reported from Germany [17]. We did not detect EV71 although this strain is reported to circulate in different European countries [18, 19]. However, cases of hand, foot, and mouth disease are typically seen by general practitioners without laboratory testing. Therefore, information on EV71 is scarce supporting the notion that monitoring of EV diversity is an important issue not only for public health. It has been shown that genotyping can help to rapidly identify novel types which are associated with severe disease [5]. Interestingly, the detection of EV and HPeV has been demonstrated in sewage samples which might serve as a method to rapidly detect spread of EV strains [19]. In addition, recombination of EV is a common event and surveillance of sewage can also detect less pathogenic EV which otherwise remain unnoticed [19].

Importantly, HPeV3 infections tended towards higher RNA concentrations compared to infections with EV although the difference was not statistically significant. This finding was also seen when only children <3 months of age where included in the analysis. Of note, both HPeV samples were obtained significantly later after symptom onset compared to EV positive samples suggesting even higher HPeV3 RNA concentrations. It is speculative if a missing RGD motif in HPeV3 and a possible neurotropism account for higher RNA concentrations [20]. A variation in virulence as proposed in a study may also be responsible [21]. Besides intrinsic viral factors recent seroepidemiological studies have demonstrated that more than 30 % of child-bearing aged women lack antibodies to HPeV3 [22]. Another study showed a low prevalence of HPeV3 specific neutralizing antibodies among the adult Dutch (10 %) and Finnish (13 %) population [23]. Of note, the authors speculated on difficulties in producing HPeV3 specific antibodies and the possibility of a test artefact. However, if the lack of passively transferred maternal HPEV3 antibodies renders neonates vulnerable to infection remains speculative. In contrast, antibodies to EV are widespread and might prevent EV replication to high titers. For EV, genotype-dependent RNA concentrations have been described recently. Volle and colleagues demonstrated higher virus concentration in echovirus 30 cases compared to those with echovirus six infection [24]. We could observe a trend to higher RNA concentrations in echovirus 30 cases but without statistical significance. Clearly, more patients are needed to confirm our findings.

Interestingly, Harvala et al. reported HPeV RNA concentrations <500 copies/ml in CSF samples of young infants submitted for bacterial sepsis screening [25]. From the same study it should be noted that spill-over of EV and HPeV, with low viral concentration in CSF, can occur from the blood and the mere detection of viral RNA in the CSF is not sufficient for a diagnosis of CSF infection, particularly if other indicative findings of CNS infection like pleocytosis are absent [25]. Unfortunately, we were not able to systematically retrieve white cell count in CSF samples as a marker for CNS infection due to the retrospective nature of the study. However, they could demonstrate higher RNA concentrations in blood samples suggesting that this type of sample should be preferred in sepsis evaluation. Choosing the best sample type for diagnosis was also addressed by another study which showed that stool samples were superior over CSF samples [26]. On the other hand, HPeV was shown to be also present in stool samples of healthy children indicating asymptomatic shedding after infection [27]. From these data it is obvious to alert clinicians that samples from multiples sites can considerably increase diagnostic yield. In our retrospective study we were not able to retrieve corresponding blood samples from our patients. However, we could show that typing was positive from stool samples in those patients in which typing from CSF samples failed for unknown reasons supporting the notion that multiple samples prove beneficial. From a technical point, the use of contemporary PCR assays is highly recommended. We could show that the detection rate for EV increase by a quarter upon use of recently published assay [10]. According to Dierssen et al. modifications of the primer and probe binding sites increased the detection of divergent echovirus 30 strains [10]. It should be noted that we might have missed cases of HPeV associated CNS infection due to low HPeV concentrations as virus concentrations below 500 copies/ml in CSF samples have been reported in infants with sepsis-like illness [25].

## Conclusions

Here, we could show that HPeV3 infection of the central nervous system occurs predominantly in young infants. Unlike for EV only one HPeV type, i.e. HPeV3, was seen. Higher viral loads in HPeV3 infection might contribute to severity of illness and deserve further studies. Beyond the detection and assessment of viral RNA concentrations genotyping might prove beneficial not only for prognostic appreciation.

## Methods

### Clinical specimens

A total of 327 archived CSF samples from 327 patients had been collected from May 1998 until October 2008. No samples were available from May to September 1999. Specimens were originally submitted to the Institute of Virology, University Medical Centre Bonn for routine virological work-up due to suspected meningitis or encephalitis at the discretion of the treating physician. Further criteria to obtain CSF samples were other neurological symptoms, e.g. meningism, bulging fontanella, seizures, reduced vigilance, and late-onset sepsis. Bacterial testing was not systematically done and thus not reported here. All samples were anonymized and stored at −20 °C. Due to prolonged storage no attempts for virus isolation were made. Residual testing was approved by the ethics committee of Bonn University Medical Centre.

### Nucleic acid extraction

For PCR samples were extracted in pools of five and tested. In case of a positive finding pools were resolved and samples were individually re-extracted and tested. Viral nucleic acids were purified by means of a viral RNA mini Kit (Qiagen, Hilden, Germany) with minor modifications. Due to little left-over of samples the input volume was set to 120 μl, and the elution volume to 100 μl.

### Real-time reverse-transcription-PCR (RT-PCR) and real-time PCR

For EV RNA detection two assays were performed as described [9, 10]. For HPeV detection the assays according to Baumgarte et al. and Nix et al. were used [28, 29]. All assays were of quantitive nature using RNA transcripts as described. In brief, amplicons of each real-time RT-PCR were cloned into pCR4 plasmid vector (Life Technologies, Karlsruhe, Germany) and in vitro RNA transcribed using the MEGAScript T7 in vitro transcription kit (Life Technologies) [28]. The detection of cytomegalovirus (CMV), herpes simplex virus 1/2 (HSV), and varizella zoster-virus (VZV) was done as described elsewhere [30–32].

### EV and HPeV typing

Typing of EV and HPeV was conducted as described [28, 33]. EV positive samples were sequenced in the VP2 gene [33]. For HPeV the complete VP1 region including the RGD motif and the VP3/VP1 junction region was sequenced according to Haravla et al. [3]. Sequencing was done on an ABI Prism 3130 using the Big-Dye Terminator kit (Applied Biosystems, Weiterstadt, Germany). Sequences were analyzed using Bio-Edit and by comparison with published sequences in NCBI GenBank.

### Statistics

Statistical analysis was performed using the GraphPad Prism 6 software package (Graphpad, La Jolla, CA, USA).
